# Older patients’ attitudes towards, and perceptions of, preoperative physical activity and exercise prior to colorectal cancer surgery—a gap between awareness and action

**DOI:** 10.1007/s00520-019-05237-7

**Published:** 2019-12-20

**Authors:** Emelie Karlsson, Oili Dahl, Elisabeth Rydwik, Malin Nygren-Bonnier, Mia Bergenmar

**Affiliations:** 1 Department of Neurobiology, Care Sciences and Society, Karolinska Institutet, Alfred Nobels Allé 23, 141 83 Huddinge, Sweden; 2 Allied Health Professionals, Function Area Occupational Therapy & Physiotherapy, Karolinska University Hospital, Stockholm, Sweden; 3 FOU nu, Research and Development unit for the elderly, Stockholm County Council, 177 21 Järfälla, Sweden; 4 Department of Oncology-Pathology, Karolinska Institutet, 171 76 Stockholm, Sweden; 5 Department of Nursing Science, Sophiahemmet University, 114 86 Stockholm, Sweden

**Keywords:** Qualitative content analysis, Qualitative research, Abdominal cancer surgery, Preoperative care, Motivation

## Abstract

**Purpose:**

Time for preoperative optimisation prior to colorectal cancer surgery is limited and older people tend to decline exercise interventions. This study sought to describe attitudes towards, and perceptions of, preoperative physical activity and exercise in older people prior to colorectal cancer surgery.

**Methods:**

This is a qualitative interview study, analysed with inductive content analysis. Seventeen participants scheduled for colorectal surgery were recruited as a purposeful sample from two hospitals in Stockholm, Sweden. Individual semi-structured interviews were conducted, face-to-face (*n* = 8) or by telephone (*n* = 9).

**Results:**

Nine participants were male, median age was 75 years (range 70–91). The theme, ‘a gap between awareness and action’, was identified based on two main categories: ‘Attitudes towards preoperative physical exercise have a multifactorial base’ and ‘Preoperative physical exercise is possible with a push in the right direction’. The material described a gap between awareness of the benefits of physical activity and reports of performing physical activity. The reasons for the gap between thoughts and action in this respect seem to be multifactorial. Support from others emerged as an important possibility for overcoming the gap.

**Conclusions:**

A gap between the patients’ awareness and action appeared in our material. Understanding this can guide healthcare professionals (HCPs) as to the support needed preoperatively. Advice on physical exercise before surgery should be specific, and individually tailored support for action should be offered. This support should also consider the individual’s current physical activity and preoperative attitude towards physical exercise.

## Introduction

Research indicates that an optimisation of physical capacity prior to surgery might decrease the risk of postoperative complications and functional decline [1–5]. Nevertheless, older people often tend to decline participation and have poor compliance with interventions involving physical exercise [6, 7]. In colorectal cancer care, the time from treatment decision until surgery can be as short as 2 weeks, limiting the time available for an exercise intervention and increasing the demands to reach sufficient exercise intensity. In a recent feasibility study of a home-based preoperative exercise programme for people over the age of 70, 65% of eligible patients declined participation, but among those who participated, the compliance to the exercise intervention was high (97%) [8].

A consensus document, incorporating experts from the *European Society of Surgical Oncology*, pointed out that multi-morbidity might lead to changes in clinical decision-making when it comes to older people with colorectal cancer [9]. In a similar study involving colorectal surgeons’ opinions on preoperative physical exercise, it was agreed that physical exercise programmes should be included in the preoperative process [10]. In studies of patients’ experiences in the period prior to abdominal cancer surgery, patients talk of a feeling of losing control, a need for information from healthcare professionals (HCPs) and a need for advice and support regardless of the preoperative timeframe [11–14].

A few studies specifically highlight preoperative physical exercise [15–18]. Mooney et al. described patients’ experiences with exercise prior to cardiac surgery, and suggest that it can be a valuable approach to meet the patients’ needs during the waiting period [15]. Burke et al. explored patients’ experiences of participating in an exercise programme prior to rectal cancer surgery, where the participants reported a feeling of being safe and encouraged, and having a sense of control [18]. In a study on participants in a prehabilitation programme prior to colorectal or lung cancer surgery, feeling physically ready for surgery was identified as a motivator [17]. However, practical issues regarding transportation posed as a difficulty. The informants in all studies were participants in exercise programmes, whereas there is no information from patients not receiving or declining participation in preoperative exercise. In this study, the aim was to describe older people’s attitudes and perceptions towards physical activity and exercise when scheduled for colorectal cancer surgery.

## Method

### Participants, sampling strategy and context

A qualitative interview study was conducted. Persons ≥ 70 years of age scheduled for colorectal cancer surgery at two hospitals in Stockholm County (Ersta Hospital and Karolinska University Hospital) were asked to participate from October 2017 at Ersta, and from February 2018 at Karolinska, until November 2018. Information regarding the study was first provided by research nurses at the hospital and then by the corresponding author by phone for oral informed consent and to schedule the interview. Written informed consent was collected before the interview started. To be eligible, the patients had to understand and speak Swedish. The exclusion criterion was a health status prohibiting physical exercise. A purposeful sample, based on age (to include a range of ages above 70) and gender, was selected to ensure information-rich participants [19, 20]. The study was approved by the Regional Board of Ethics in Stockholm (Dnr: 2016/1587-32, and 2017/1246-32). Interview transcripts are stored in a locked cabinet at the research unit. Research subjects were encoded in chronological order. The code keys are kept separate from the audio recordings and transcribed text material. The interview questions could have been perceived as an intrusion into the private sphere, but could also be seen as an opportunity for the informants to express themselves and describe their situation through participation in the study.

### Data collection

The sample size was based on obtaining sufficient information power to achieve the aim of the study [21]. As nothing notably new related to the aim appeared, we chose to finalise the data collection at seventeen interviews. Semi-structured interviews were conducted individually at the end of the waiting period, i.e. close to the surgery. The interviews were audio recorded with a dictaphone. Eight interviews were conducted face-to-face in the participants’ homes (*n* = 2) or at the hospital ward (*n* = 6), as the participant preferred. Nine interviews were conducted by telephone due to participants living in another county or participants perceiving that they did not have time for a face-to-face interview. Two interviewers (EK and OD) conducted ten and seven interviews, respectively. One interviewer was a doctoral student and physiotherapist (EK), and the other a PhD and nurse (OD). The physiotherapist had clinical experience of the topic and research experience with the patient group. The nurse had experience in interviewing techniques, with the patient group, and was informed about the content of a possible exercise intervention. As a support for the interviewers, an interview guide was created by EK, OD, MB and MNB (Table 1).

**Table 1:** Main interview questions (translated from the Swedish interview guide)

Area 1: Preoperative physical exercise	
A. How do you view physical exercise?	
B. What do you think about exercising while waiting for your surgery?	
How have you changed, or not changed, your physical exercise/activity before your surgery?	
What impact do you think preoperative exercise has on recovery after surgery?	
What barriers and/or possibilities do you see to performing physical exercise before surgery?	
Area 2: An alternative preoperative pathway	
A. What are your thoughts about exercising with a physiotherapist while waiting for your surgery? At home?	
What are your thoughts about extending the waiting time (by approximately 1 week) to be able to exercise more before the surgery?	
Debriefing	
Do you have any additional thoughts regarding this that you want to mention?	

### Interview guide

The guide was based on two topics: ‘preoperative physical exercise’ and ‘an alternative preoperative pathway’. Before the interview, the aim was briefly explained and the participant’s rights were repeated. It was made clear to the participants that they were the experts and we wanted to understand the topic from their perspective. Clarifying and follow-up questions were asked when appropriate, for example ‘You said…what did you mean when you said that?’, ‘Can you elaborate?’ and ‘Can you give an example?’. The duration of the interviews ranged between 16 and 30 min, and they were transcribed verbatim. Demographic data such as living situation, comorbidities, previous surgeries and cancer stage was collected from the medical records and patient interviews. Patients were asked to complete the questionnaire physical activity scale for the elderly (PASE) after the interview to collect data on physical activity level.

### Analysis procedure

Content analysis was performed [19] using the inductive approach defined by Elo and Kyngäs [22]. All interviews were transcribed by the corresponding author and read by everyone involved in the analysing procedure (EK, MB and MNB). From the transcripts, meaning units related to the study objective were identified. Second, open coding was conducted in writing on the transcripts and later grouped in a coding sheet. The initial extraction of meaning units and coding followed the text as closely as possible and used an iterative process going back and forth between condensed meaning units and codes. The first steps were conducted by the corresponding author and verified by colleagues with experience in content analysis (MB and MNB) to check for consistency between the authors. When consensus was reached, the codes were grouped into possible sub-categories, and further discussions and revisions were made jointly in the group, resulting in nine sub-categories. In a final group discussion (EK, MB and MNB), categories with content-characteristic words were abstracted and staged within two main categories. In the result section, some interpretations were included, resulting in a theme. Table 2 shows an example of the analysis procedure.

**Table 2:** Scheme of analysis (example from text unit to main category)

Text unit	Condensed meaning unit	Code	Sub category	Category	Main category
‘I think it would be more effective if someone nags you. Absolutely. I can only relate to myself, and I am not unique, ehh… I think that’s the way we work, there are probably very few who are so energetic that they can do it a hundred per cent on their own.’ – P8	It is more effective if someone nags you; few are energetic enough to make it on their own.	Need of encouragement	Need of psychosocial support	Support to enable action	Preoperative physical exercise is possible with a push in the right direction

## Findings

**Fig. 1:**
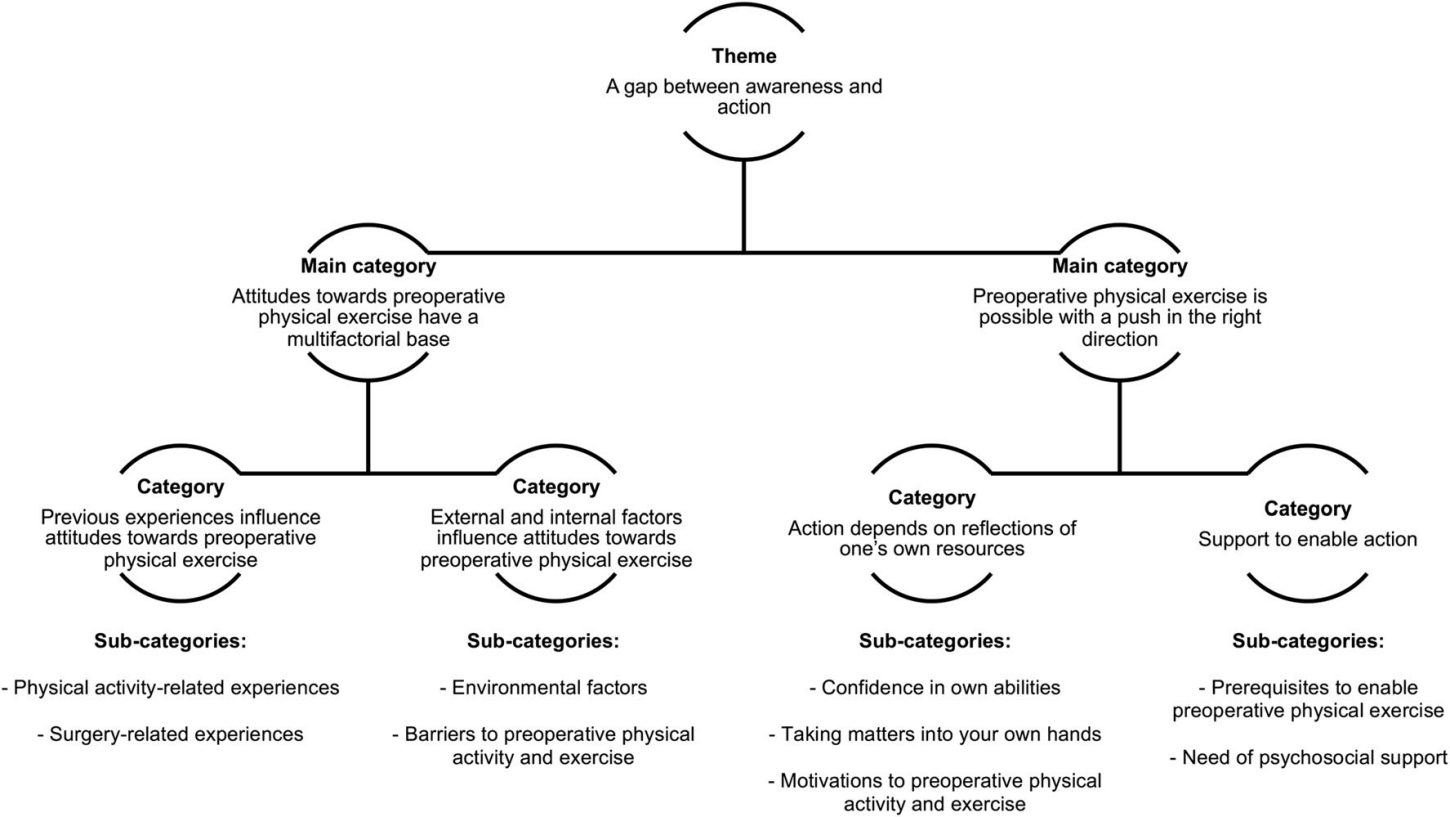
Overview from sub-categories to theme extracted from the interviews

Table 3 shows data on the seventeen participants. The theme ‘A gap between awareness and action’, the two main categories ‘Attitudes towards preoperative physical exercise have multifactorial base’ and ‘Preoperative physical exercise is possible with a push in the right direction’ and the four categories building up the main categories are presented in Fig. 1.

**Table 3:** Description of the participants interviewed (*n* = 17)

Variable		Value
Age, median (range)		75 (70–91)
Sex, *n*	male:female	9:8
Living situation, *n*	With partner	11
	Alone	6
Education, *n*	Compulsory level	4
	Above compulsory	5
	University	8
Cancer type, *n*	Colon	10
	Rectal	7
Charlson comorbidity index, median (range)^a^		2 (0–6)
Neoadjuvant treatment, *n*	None	14
	Radiation therapy	2
	Chemotherapy	1
Previous surgery, *n*^b^	No	4
	Yes	13
Self-reported preoperative physical activity level, median (range)^†^	Total	102 (15–179)
	Males	120 (15–146)
	Females	101 (40–179)

Normal values for self-reported physical activity: 70–75 years males 102.4, females 89.1; 76–100 years males 101.8, females 62.3 [23]

^a^Not adjusted for age

^b^Pancreatic surgery, splenectomy, prostatectomy, appendectomy, hepatectomy, hysterectomy, pacemaker, surgery, CABG (coronary artery bypass graft), sectio, hip replacement, rectal resection, urinary tract surgery, cystectomy, knee surgery or tonsillectomy

^†^*n* 16, one missing questionnaire

### Theme: a gap between awareness and action

The participants’ self-described awareness of possible benefits from being physically active and the motivation for physical activity were strong. Conversely, the material also described a gap between this awareness and actually engaging in physical activity. The reasons for this gap were described as caused by multiple factors before and during the preoperative period. Help and support from others were mentioned as possibilities for overcoming the gap.

#### Main category: attitudes towards preoperative physical activity and exercise have a multifactorial base

Attitudes towards preoperative physical exercise have a multifactorial base, where various factors work as facilitators or inhibitors to preoperative physical exercise. These factors can create a feeling of hope if they are modifiable or dejection if they are non-modifiable. The factors arise from past experiences as well as present aspects in the environment and preoperative context.

##### Category: previous experiences influence attitudes towards preoperative physical exercise

This category implies that previous experiences, regarding both physical activity throughout life and previous surgery, mirror patients’ attitudes towards physical activity and exercise in the preoperative context. The participants expressed views ranging from physical exercise being a natural part of life to being a pure necessity for staying healthy. When it was considered a natural part of life, and if physical exercise had previously resulted in positive feedback, the patients also expressed an openness towards conducting preoperative physical exercise. Conversely, negative experiences with physical exercise seemed to be associated with a more restricted interest in conducting preoperative physical exercise.

‘You should really move all the time, shouldn’t you ... not just before an operation. I think that’s the most important thing. Nothing has changed because I am undergoing surgery, I have kept active’ – P5

‘Physical exercise does not sound appealing … I am not really into gymnastics at all, they ruined my taste for that in school.’ – P4

If they had had previous surgery, their expectations of the current surgery were related to the outcomes of the previous one. If postoperative recovery was a positive experience, the same result was expected this time despite a different context and health status. That mindset was associated with a feeling of being ready for surgery without any additional effort.

‘I don’t think I’ll have any issues because I have had major surgery before … more than ten years ago.’ – P2

##### Category: external and internal factors influence attitudes towards preoperative physical exercise

Environmental factors, including physical, social, attitudinal or psychological environment, were described as influencing preoperative physical exercise in either an inhibiting or facilitating way. The participants spoke about limitations in the environment, being dependent on healthcare resources to enable exercise, changes in life such as moving from rural to urban areas and taking care of the household alone and thus feeling they had no time for exercise. Support from next of kin or friends was considered a facilitator for activity.

‘Normally I live at our summer house since my retirement, where I always have on-going projects. Since last summer when this started, I haven’t been there. Which is a huge change for me because there I’m active all the time, but here in the city, there is nothing to work on.’ – P6

‘A fear that I cannot walk alone. Even if I have the walker, I have to have someone with me ... and I don’t think that home care and the doctors would allow me to go out alone. It makes it more difficult because I have to have someone from home care that can accompany me and wait for me… and sometimes they are short of staff.’ - P16

Various forms of barriers were described as obstacles to performing physical activity and exercise. This sub-category included mental barriers such as doubting the effectiveness of a short-term preoperative exercise programme, stating that it was ‘for others, not for me’, and feeling uncertain of the outcome. Patients also expressed conflicts between a social desirability to be active and human laziness. In addition, physical barriers related to cancer symptoms or treatment as well as comorbidities or earlier impairments were further hindrances.

‘… I got tired back in February without knowing why, which resulted in reduced ability to do things I normally do. One might blame age, but it was not age, it was the disease.’ – P15

This category also included a description of competing focuses and other priorities—having a different focus of interest than physical activity during the preoperative period. Conflicts with other examinations and a busy schedule in combination with a short time frame were competing factors they mentioned. The perception that the surgery was urgent led to the patients reporting that taking time for optimisation was secondary. Another central focus was the postoperative period, where they described a need to conserve their energy until after the surgery.

‘It has been difficult to get started on anything at all, because first I went for a colonoscopy and then there was some X-ray ... and then I had a colonoscopy again the other day... there have been so many activities involving the… so I don’t think I could have done any exercise in-between.’ – P9

‘My body will obviously react (to the operation)... and as I told you, after the surgery ... I need the energy to recover. That’s when it is needed.’ – P7

#### Main category: preoperative physical activity and exercise is possible with a push in the right direction

The motivation to engage in preoperative physical activity and perceptions of exercise as a way of preparing for surgery and regaining their abilities postoperatively were raised. Nevertheless, the interviews also revealed a conflict between that motivation and the actual reported physical activity level. The participants described prerequisites they needed to initiate physical exercise on their own or with help from others, such as active support, including tools to perform the relevant exercise, encouragement and monitoring from HCPs, guidance, structure, accessibility and time set aside for exercise.

##### Category: action depends on reflections of one’s own resources

This category describes the impact of confidence in one’s own abilities. Participants who had a high belief in their own abilities—such as feeling physically strong, not letting impairments inhibit activity and having a positive self-image—also had a positive attitude towards taking action to be active. In contrast, having self-doubt, as well as expressing age-related and exercise limitations, seemed to be related to less interest in being active prior to surgery.

‘I have a house to maintain, a garden to take care of and two dogs to look after. That is enough for me at this age.’ – P4

‘You can see yourself as a sick old person … and if that is my self-image I’ll adapt to that role and be a sick old person. I can also see myself as a healthy, strong person who likes being physically active and being outside, and if you have that self-image I think you have better chances of getting back to normal life again.’ – P1

Some also described taking matters of physical exercise into one’s own hands by taking the initiative to exercise, increasing exercise discipline and the gradual progress of preoperative activity to be fit for surgery. This included adapting to current circumstances and creating one’s own coping strategies such as adding exercises to previously experienced programmes.

‘Physical exercise makes me feel good. Nowadays, when I do not have a physiotherapist to lean on, I will have to do it myself. That works … for me at least.’ – P5

The biggest motivation and drive for the participants to pursue preoperative physical activity or exercise was an aim for postoperative independence. These participants felt that a strong body was equal to a strong recovery and an improved healing process, and in contrast, they emphasised the risks of being inactive. Some also mentioned that engaging in physical exercise during the waiting time could reduce sad thoughts related to the diagnosis and upcoming surgery and create meaning during an otherwise mentally stressful time.

‘You know, the wait is always hard; it’s hard to wait for something. So there should be some meaning to the wait. It should motivate you, like “I’m doing this so my body will work as well as possible both before and after surgery.” That would be good.’ – P15

##### Category: support to enable action

The need for active support to be able to conduct preoperative exercise was repeatedly expressed. Among these were practical support and prerequisites such as individual exercise guidance and instructions from a professional to achieve effectiveness, theoretical knowledge and practical tools from various fields of healthcare. They also brought up psychosocial support such as help from others, need of a nudge and need of encouragement.

‘You might need a nudge, someone telling you what to do. My experience is that no one has done that at all. It’s like you need to have more motivation when you’re sick than when you’re well.’ – P6

‘I know, but many times knowing is not enough.’ – P4

This category describes a lack of communication from HCPs regarding preoperative physical exercise and activity, which was associated with a feeling of not knowing what to do or change. If they received exercise information, it was considered too general and unspecific.

‘I didn’t get anything… the nurses only said, “take walks”. Nothing special I was supposed to do, just go out walking.’ – P12

‘On the other hand, I might point out that no one has talked to me about exercise since this started. No one has said a word about it. At the same time I have my own responsibility to figure that out, but on the other hand human beings are quite lazy.’ – P6

However, the participants expressed a sense of feeling cared for during the preoperative period concerning general care, as well as a trust in HCPs.

‘I see exercise positively ... I absolutely do. But it seems that I am fit ... this morning a cardiac doctor called and said that everything was fine … so I am ready for surgery.’ – P2

## Discussion

This study provides a deeper understanding of patient attitudes towards, and perceptions of, preoperative physical activity and exercise—whether or not they want to participate in a preoperative exercise programme—in older people scheduled for colorectal cancer surgery. Overall, our findings describe a gap between awareness and action, including multiple factors creating this gap and the need to diminish the gap and possibly enable preoperative physical activity.

Previous studies have demonstrated that physical activity in older adults is determined by multiple factors, which we identified in the preoperative context as well [24–26]. Our findings also indicate that social environment affects attitudes towards preoperative physical activity. Older adults with better social support, linked to physical activity, from family members are more likely to engage in physical activity in their leisure time [27]. Studies exploring older adults’ perspectives on physical exercise participation found, somewhat similarly to our findings, the themes ‘social influences; physical limitations; competing priorities; access difficulties; personal benefits of physical activity; and motivation and belief’ [28]. However, our findings add the influence of previous surgery-related experiences, time constraints and physical as well as psychological limitations from cancer treatment and diagnosis. This makes the patient perspective on preoperative activity and exercise a somewhat more complex matter. In previous studies, participants describe mainly positive aspects regarding preoperative physical exercise. However, the results are based on those who participated in the programmes [15, 16, 18], thus missing the attitudes of the patients who declined exercise during recruitment.

Physical activity is defined as ‘any bodily movement produced by skeletal muscles that results in energy expenditure’, and physical exercise as ‘planned, structured, and repetitive bodily movement done to improve or maintain one or more components of physical fitness’ [29]. As physical exercise is a subset of the broader concept of physical activity, we included the participants’ perceptions both in regard to physical activity and physical exercise. Previous physical activity-related experience seemed to define how the participants in this study conceptualised physical exercise and activity, as this differed between individuals. Physical activity and exercise were described in such varying ways as going to the gym or being engaged in everyday activities such as dog walks and household chores. The participants tended towards activities and exercises that felt familiar. One possible reason for this might be a lack of knowledge regarding relevant physical exercise for the preoperative period and the possible benefits it may provide. Therefore, it is important to inform the patients regarding the type, dose and progression of exercise that is sufficient preoperatively, as well as to go from general to more tailored advice [30]. Additionally, the perception of the concept might also differ between HCPs mediating the preoperative information.

The category ‘support to enable action’ in our findings described a lack of communication from HCPs regarding physical exercise in the preoperative period. In general, patients request structural and individualised information from HCPs prior to surgery, and not solely regarding physical exercise [12]. It has been shown that patients at high risk tend to underestimate their risk of postoperative morbidity, but when made aware, the majority would consider taking action to preoperative optimisation [31]. Clinician-patient communication has shown to have positive effects on outcomes such as patient knowledge and understanding, adherence and better self-care abilities [32]. Prehabilitation is an emerging area of research globally, and several studies have reported positive results on preoperative physical capacity [3, 4] and some on postoperative outcomes [1, 2]. Recently, suggestions on clinical pathways within the area have been proposed [5, 30]. Still, in many countries and in Sweden, rehabilitation in perioperative care has been centred on the postoperative period in clinical practice, while prehabilitation is currently not yet implemented as a part of routine practice [33]. Therefore, a preoperative focus to enable prevention may be a new perspective for the patient. One suggestion is to redistribute the focus that is now fully on postoperative action and create a shared focus with the opportunity to prepare for surgery.

The category ‘Action depends on reflections of own resources’ described the impact of confidence in one’s own abilities on attitudes towards preoperative physical activity and exercise. Previous studies have demonstrated independent associations between beliefs in one’s own capacities or self-efficacy and physical activity [34]. It has been suggested that a combination of self-efficacy and a sense of control might improve individual resources and in turn, the likelihood of taking action for one’s own health [35]. Furthermore, action planning has proven to be a determinant of initiation of physical activity [36].

Even though preoperative physical activity and exercise occur in a specific context, it is based on physical movement as described in the Movement Continuum Theory (MCT) by Cott et al. and later by Allen [37, 38]. One of the overall principles of the MCT is ‘movement is influenced by physical, psychological, social and environmental factors’, which also holds true in our findings [37, 39]. Within the MCT, physical activity and exercise are seen as tools for enhancing movement capacity and decreasing the gap between current and preferred movement capability. In the preoperative context, the task of the physiotherapist may be to give the patient tools to achieve efficiency, make modifications and provide a bridge to overlap the gap between awareness and action.

### Methodological considerations

There was a risk that the participants would feel a social desirability to answer questions regarding physical activity in a certain way due to the professions of the interviewers. To reduce that risk, the participants were informed at the beginning of the interview that they were the experts and we aimed to describe the topic with as much information as possible. Having two interviewers with different professional backgrounds may have created diversity in the content of the interviews. The interview guide helped to keep the interviews focused around the topic and to prevent superfluous information, and to prevent the interviewer from becoming a co-author during the interview [40]. In addition, the group involved in the analysing procedure included two physiotherapists and one nurse.

The word (prolonged) waiting time, as used in the interview guide, may itself create negative associations. Changing this vocabulary to ‘preparation time’ might improve acceptance. Some patients declined participation in this study due to lack of time prior to surgery. Including those patients might have provided more reasoning regarding barriers related to the short time frame of the preoperative period. Given the complexity of collecting data during the short and busy preoperative time frame, a flexibility regarding the location of the interviews was needed. Therefore, the participants themselves chose the location of the interview (at home, by telephone or at the ward on admission). Half of the interviews were telephone interviews, which lack in visual or nonverbal data compared with face-to-face interviews and may in turn lead to less information and participant involvement [41, 42]. In the context of this study, the interviewers perceived that the participants felt less stressed when being able to conduct the interview without any additional transportation to the hospital. In addition, when reviewing the transcripts, the extent of information was comparable between the two different interview methods. As attitudes towards and previous experiences of physical activity may differ with age and gender [43], we used purposeful sampling. We believe that the results are transferable to contexts with similar preoperative care processes (in relation to time and clinical praxis) and older people scheduled for cancer surgery.

## Conclusion and implications

This study demonstrated a gap between the patients’ awareness and actions, which HCPs need to be aware of. The clinical implication of the findings would be to identify the factors, beyond physical performance, for each individual and use that knowledge to support and tailor an individualised preoperative pathway. General advice on physical activity before surgery should be replaced by more specific advice and support for action, tailored to the individual patient and taking into account the individual’s current physical activity level and attitude towards physical exercise in the preoperative period.
